# 

**Published:** 1914-12-19

**Authors:** 


### CONTENTS.

| | PAGE |
|---|---|
| **Christmas Day 1914** ... ... ... ... ... | 253 |
| **Some General Influences of the War** ... ... | 253 |
| **Hospital and Institutional News** ... ... ... | 255 |

Our Christmas Appeal Number—Our War Correspondents and Christmas Week—The Poor-Law Infirmary on the Eve of Change—The Voluntary Spirit in Hospitals and Armies—The Insurance Act and Hospital Beds—Hospitals and Panel Doctors—A Doctor Dispatch-Rider—Can a Panel Doctor's Fees be Attached?—St. Katharine's Hospital Reform Scheme—The New Chairman of Leicester Royal Infirmary—A Suggested Home for Domestic Servants—An Isolation Hospital for Dublin—Refusal to Increase a Medical Superintendent's Salary—General Post of Medical Superintendents—A Scottish Training School for Institution Officials—A Dangerous Unqualified Doctor—Medical Treatment and Poor-Law Disqualification—Hospital Cheer and a Quiet Christmas—Hospital Poems—Panel Practitioners and Tuberculous Patients—City of Cardiff Hospital for the French Army—The Local Government Board and Salaries—Laymen and the Hospital Spirit—A Naval Surgeon's Life at Sea—Florence Nightingale's Hospital Letters—This Week's Drug Market.

[*See over.*]

### CONTENTS—(*continued*).

PAGE

**Surgical Experiences in the Present War** ... 261
Modern Bullet Wounds of the Soft Parts.

**The New Pharmacopœia** ... ... ... ... 262

**Two Months of War Work at Manchester** ... 263
The Royal Infirmary's Aid for the Wounded.

**Work for the Wounded of the Allied Armies** 264
Cardiff: Soldiers' Praise for the Transport Arrangements—Christmas in the Base Hospitals — St. Bartholomew's Hospital, Rochester — Doncaster: First Wounded Soldiers Arrive — Sheffield: Over 2,000 Soldiers Treated.

**Impressions of a German Hospital** ... ... 265
The Eppendorf Hospital in Hamburg.

**The London Panel Committee** ... ... ... 266

An Important Series of Decisions.

**National Insurance Act Developments** ... 267
Effects of the War on National Insurance.

**Round the World** ... ... ... ... ... ... 269

Fellow-Workers and the Roll of Honour.

PAGE

**Misconceptions** ... ... ... ... ... ... ... 270
Proposed Women's Voluntary Hospital Brigade.
A Women's Volunteer Reserve.

**The Editor's Letter-Box** ... ... ... 271
The Need for Young Qualified Medical Men.
Special Hospital for Officers Suffering from Shock—Rejoinder to Dr. Ash.
Boots for the Belgian Wounded.
The European War Fund Formed by the St. John Ambulance Department.

**Gravesend Hospital and the War** ... ... 272
Mr. W. Pearson and Miss Davidson on its Work.

**Personalia** ... ... ... ... ... ... ... 274
**Gifts and Bequests** ... ... ... ... ... ... 274
**Buildings Contemplated** ... ... ... ... ... 274
**Institutional Needs** ... ... ... ... ... ... 274
**The Institutional Worker** ... ... (Suppl.) 1
The Pasteurisation of Milk.
Answer to November Question.

